# Monodisperse thiourea functionalized graphene oxide-based PtRu nanocatalysts for alcohol oxidation

**DOI:** 10.1038/s41598-020-64885-6

**Published:** 2020-05-08

**Authors:** Esra Kuyuldar, Su Selda Polat, Hakan Burhan, Sibel Demiroglu Mustafov, Aysegul Iyidogan, Fatih Sen

**Affiliations:** 1 Sen Research Group, Department of Biochemistry, Faculty of Arts and Science, Dumlupınar University, Evliya Çelebi Campus, 43100 Kütahya, Turkey; 2 Department of Chemistry, Faculty of Science and Arts, Gaziantep University, Gaziantep, Turkey

**Keywords:** Catalysis, Energy

## Abstract

Addressed herein, thiourea functionalized graphene oxide-based PtRu nanocatalysts (PtRu@T/GO) has been synthesized and characterized by several techniques and performed for methanol oxidation reactions as novel catalysts. In this study, graphene oxide (GO) was functionalized with thiourea (T/GO) in order to obtain monothiol functionalized graphene and increase the stability and activity of the nanocatalysts. Raman spectroscopy, X-ray photoelectron spectroscopy (XPS), X-ray diffraction (XRD), TEM (transmission electron microscopy) and high-resolution transmission electron microscopy (HR-TEM) were used for characterization of the prepared nanocatalysts. The results obtained from these techniques showed that the prepared nanocatalysts were in a highly crystalline form, well dispersed on T/GO, very small in size and colloidally stable. The average size of the synthesized nanocatalysts determined by TEM analysis was found to be 3.86 ± 0.59 nm. With HR-TEM analysis, the atomic lattice fringes of the nanocatalysts were calculated to be 0.23 nm. After the full characterization of the prepared nanocatalysts, they were tried for the methanol oxidation reaction (MOR) and it was observed that 97.3% of the initial performance was maintained even after 1000 cycles while exhibiting great catalytic activity and stability with the help of T/GO. Thus, the arranged nanocatalysts displayed great heterogeneous catalyst characteristics for the methanol oxidation response.

## Introduction

The direct methanol fuel cells (DMFCs) have superior properties among reliable and long-lasting portable power sources used in devices such as mobile phones, computers, etc. Even though there are substantial improvements in DMFC systems over the last decade, more effort is needed to commercialize DMFCs by producing durable, low cost and lower size devices. Up to now, the many present nanocatalysts have been developed as electrocatalysts for DMFCs, but it is really important to obtain the optimum supporting agents that enhance the interaction and the catalytic activity between the support material and the metal catalyst^1–6^. As a catalyst support material, carbon derivatives have been commonly used^7–12^. The results of intensive studies on carbon-containing materials revealed some significant information about the catalytic activity and supporting agents^13–15^. The synthesis of nanomaterials is very important in the use of DMFCs. Specifically, nanocatalysts containing carbon-based materials^16–21^ such as carbon nanohorns, carbon nanofibers, carbon nanotubes, and carbon nano-coils have attracted attention. When compared to traditional materials, carbon-based materials have unique advantages^22–26^ such as high corrosion resistance, better electrical conductivity, and less catalyst poisoning^27–29^. The electrocatalyst based on these carbon-based materials used in fuel cells must have some desirable features such as composed of reactants facilitating reactions, controllable suitable particle size, etc.

Various methods have been used to prepare surface-functionalized carbon-based nano-catalysts^22,23,30–32^. Therefore, various catalysts^33–42^ such as Pt and Ru based have been used as electrocatalysts (PtRuWC, PtRuIr, PtRuCo, PtRuP, PtRuSnW, and PtRuRhNi). Among those, PtRu based catalysts have been extensively used for the catalytic reaction in the anode of DMFC due to their long life and the suitable surface. However, there are various problems to be overcome for these types of catalysts. For instance, insufficient durability, inactivity, crossover problem and dissolution^38,43–50^ are essential problems related to carbon-based PtRu catalysts. In order to solve those types of problems, some studies have been performed with the help of the functionalization of carbon-based materials, etc.^51–56^. Consequently, graphene oxide was functionalized mainly with different functional groups containing heteroatoms for improving the physical and chemical properties of graphite^38,47–51,57–61^.

**Figure 1:**
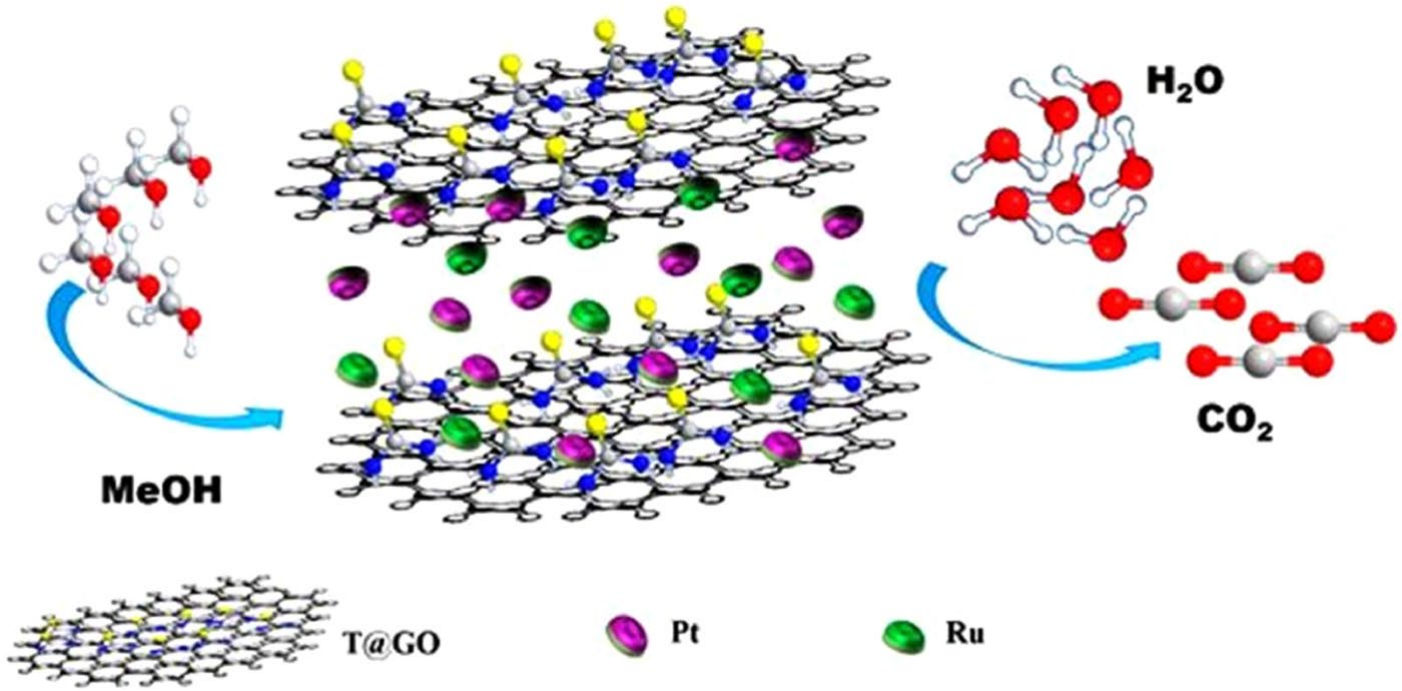
Schematic illustration of PtRu@T/GO nanocatalysts for methanol oxidation.

In this study, we have investigated the thiourea based graphene oxide (T/GO) as a potential supporting and stabilizing agent. Functionalization of graphene oxide with thiourea (T) ensures diversified potentialities to enhance the usage of graphene and increase the chemical conversion to graphene. This eliminates its poor solubility and difficult processability in both water and organic solvents make it one of the ideal materials for MOR. Here, we report for the first time that we used the thiourea graphene oxide-supported PtRu nanocatalysts (PtRu@T/GO) as the anode catalyst in DMFCs and the activity of the catalyst was enhanced due to acquiring high active sites, solubility and functionalization. Schematic illustration of PtRu@T/GO nanocatalysts for methanol oxidation was shown in Fig. 1.

## Experimental

### The procedure of preparation PtRu@T/GO nanocatalysts

To obtaining graphene oxide (GO) nanosheets from graphite, the modified Hummers’ method was carried out as shown in supporting information in detail^62–64^. Moreover, then, 50 mg of obtained GO nanosheets were dispersed in a round-bottom flask contained 10 mL THF and 1 mg/mL thiourea (T). This mixture then respectively stirred for 1 hour and ultrasonicated for another 1 hour at room temperature. The prepared solution was filtered to obtain the dark brown material apart from the solution. The dark brown graphene oxide slurry washed with EtOH to get the T/GO nanosheets neatly and then it was dried at 50 °C in a vacuum oven overnight. Under sonication, 25 mg of PtCl_4_, 25 mg of RuCl_3_ and 50 mg of T/GO were mixed thoroughly in deionized water. The mixing protocol was continued at 55 °C for 12 hours. In the next step, 100 µL of DMAB solution was added dropwise with stirring over 5-minute intervals. After all the processes, washing with deionized water was carried out. Finally, the PtRu@T/GO nanocatalysts was left to dry in the vacuum oven.

Preparation of nanocatalysts sample was performed with a solution containing 0.5 mg. mL^−1^ ethanol and copper grid (carbon covered 400 mesh), resulting mixture were evaporated. Samples were morphologically examined by taking TEM images with a JEOL 200 kV instrument. The removing excess mixture was done by using adsorbent paper, and the resulting solid sample was dried at 298 K. To get an overall analysis of PtRu@T/GO nanocatalysts almost 300 particles were investigated. XPS analysis was utilized to examine the oxidation state of the metals in the nanocatalysts as well by Specs spectrometer (1253.6 eV, 10 mA). XPS analysis was performed with Gaussian function and C 1 s line at 284.6 eV taken as reference points. XRD analysis was executed to represent the composition of PtRu@T/GO nanocatalysts by Rigaku diffractometer, X-ray generator with Cu K radiation at 40 kV, 40 mA.

### The activities of electrochemical nanocatalysts

After full characterization of the prepared nanocatalysts, the catalytic activities of the electrochemical catalyst were performed by a chronoamperometry (CA) (Gamry, Reference 3000) and cyclic voltammetry (CV). The three-electrode system consists of a working electrode, a counter electrode, and a reference electrode. These were a glass carbon electrode (GCE) covered with thin the catalyst, Ag/AgCl, and Pt wire, respectively. An electrolyte containing potassium hydroxide (0.5 M), methanol (0.5 M) and saturated nitrogen gas at room temperature was used to perform CA and CV analysis. In the beginning, the samples were activated in a nitrogen-saturated potassium hydroxide (0.5 M), a voltage in a range of -0.9 + 0.2 V, by CV at a rate of 50 mV/s.

## Results and discussion

### Characterization of the PtRu@T/GO nanocatalysts

**Figure 2:**
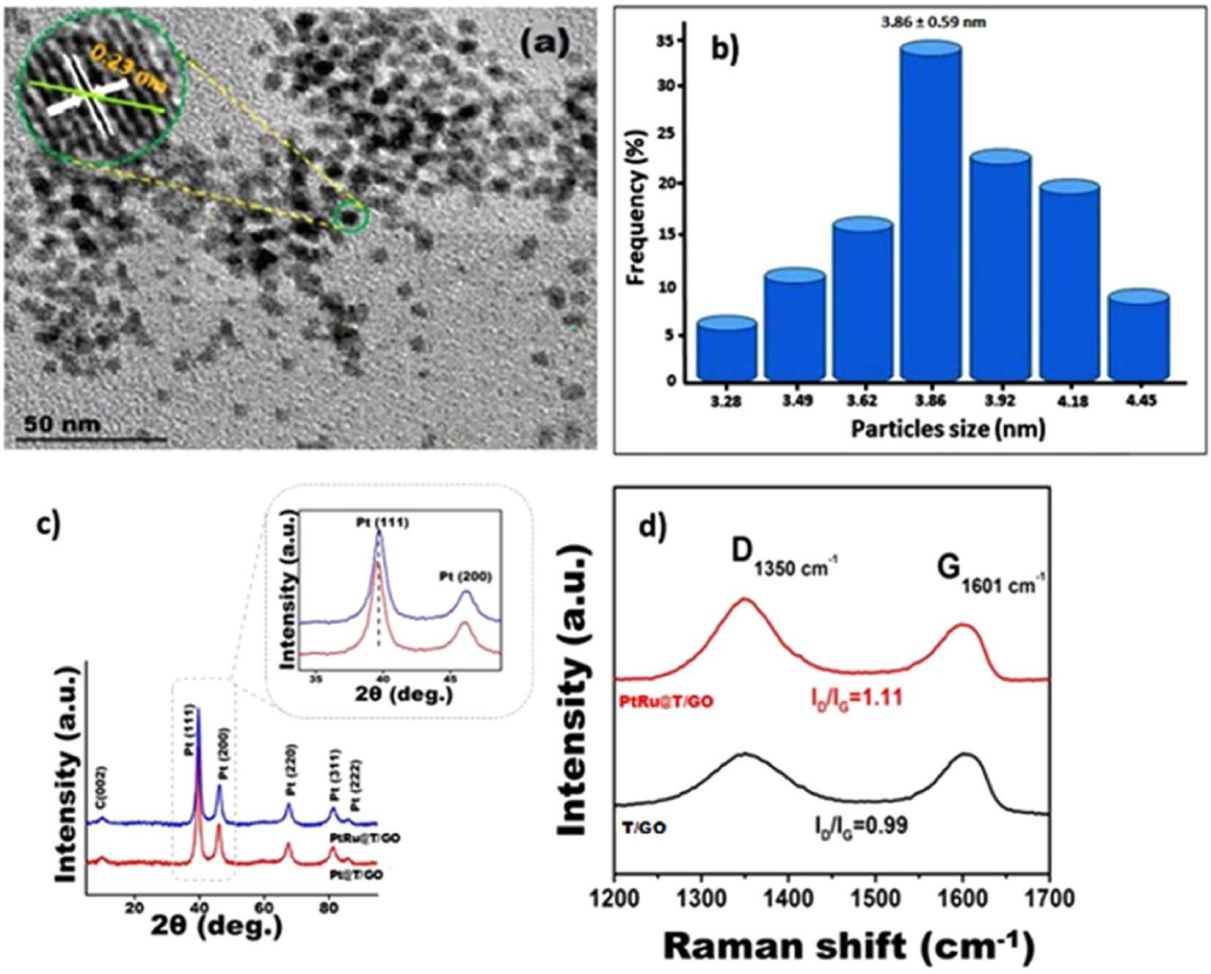
(**a**) TEM image of as-prepared PtRu@T/GO nanocatalysts indicating excellent catalyst morphology and (**b**) histogram, (**c**) X-ray diffraction pattern of as-prepared Pt@T/GO and PtRu@T/GO nanocatalysts. (**d**) Raman analysis of prepared materials.

Various analytical methods like HR-TEM, TEM, XPS, Raman spectroscopy, and XRD analyses were carried out for illuminating surface properties and morphology, chemical, and physical structure of the current nanocatalysts. For instance, the TEM analysis images are shown in Figs. 2a and S1 and they revealed that the composition of the PtRu@T/GO nanocatalyst was homogeneous, and the mean diameter of the particles was found to be 3.86 ± 0.59 nm (Fig. 2b). Also, these findings according to TEM analysis during the formation of PtRu@T/GO nanocatalysts showed that no agglomeration was detected, and obtained nanocatalysts were spherical. HR-TEM analysis also indicated that the atomic lattice fringe of particles was calculated as 0.23 nm which is consistent with the data in the literature^65–69^. Further, XRD analysis was used to examine the crystal structures of the bimetallic PtRu@T/GO nanocatalysts synthesized homogeneously, and the crystal structure was compared with the crystal structure of Pt and Ru. As shown in Fig. 2c, XRD patterns of Pt@T/GO and PtRu@T/GO were examined in order to see the crystalline structure of the catalysts. As seen in the model, monodisperse PtRu nanocatalysts were found to be in the face-centered cubic (fcc) structure in the XRD model, and in this structure, five characteristic peaks are corresponding to the (111), (200), (220), (311) and (222) planes respectively for the PtRu nanocatalysts in the bimetallic structure. Besides, a peak at 12.5^o^ is defined as T/GO, and according to data in Fig. 2b, a slight shift of 2θ values of bimetallic PtRu@T/GO nanocatalysts compared to the monometallic ones shows the alloy formation of prepared nanocatalysts.

The Scherrer Eq. (1) was utilized for calculating the average size of PtRu@T/GO nanocatalysts, and it is determined as 3.86 nm^70–72^ which is consistent with TEM analysis.

1 $$d({\rm{\AA }})=\frac{k\lambda }{\beta cos\theta }$$

where k is a coefficient (0.9), β = a half maximum diffraction peak, θ = the angle at the position of peak maximum (rad) λ = X-ray wavelength (1.54 Å). Platinum diffraction peak (111) was used to calculate the lattice parameter values as 3.92 Å.

**Figure 3:**
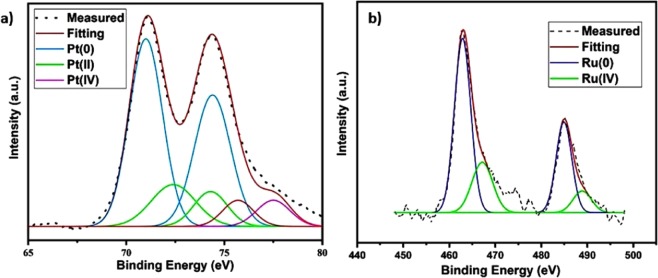
(**a**) 2D X-ray photoelectron spectra of Pt 4f and (**b**) 2D of X-ray photoelectron spectra of Ru 3p in PtRu@T/GO nanocatalysts.

Raman spectroscopy was also used in order to determine the ratio of D and G bands of prepared materials as shown in Fig. 2d. The defects on the graphene assess the ratio of peak intensities for the D and G bands (ID/IG). This ratio is 0.99, and 1.11 for T/GO, and PtRu@T/GO nanocatalysts, respectively. The slightly higher degree of defects (D/G ratio) on PtRu@T/GO nanocatalysts compared to the T/GO can be explained by the functionalization of T/GO. Moreover, electronic properties, elemental structure and chemical oxidation of the metals in the PtRu@T/GO were detected by XPS analysis. In XPS analysis, ruthenium 3p and platinum 4f orbital regions were investigated; therefore, the XPS peaks were fitted to the Gaussian method and calculated with the help of the integration area of each peak. C 1 s peak at 284.6 eV was taken as a reference^73–77^ for the accuracy of binding energies according to the XPS spectrum data. Experimental binding energies (Fig. 3) of ruthenium and platinum were compared to the binding energies exist in the literature. The obtained experimental binding energies for ruthenium and platinum are observed in 462.3 eV, and 70.2 eV, respectively. When the experimental data compared to the data exist in the literature, XPS analysis demonstrates that the surface of PtRu@T/GO has covered mostly with metals and unoxidized species. The presence of a small energy change for ruthenium at 3p_3/2_ indicates the formation of PtRu@T/GO nanocatalysts. Moreover, it can be stated from the experimental data in Fig. 3 that the composition of the nanocatalysts is mostly metallic due to the species of platinum (0) and ruthenium (0). Besides, there are some other peaks related to the oxidized species such as Pt (II) and Ru (IV) ions due to oxidation, as seen in Fig. 3. The peak region of platinum is greater than ruthenium since the higher sensitiveness of Pt 4f compared to the ones of Ru 3p. O 1 s XPS spectrum of PtRu@T/GO nanocatalysts (Fig. S2) displays that C-O and C=O bonds become mostly prominent while the other oxygen groups have decreased to minimum amounts as given in supporting information in detail.

### Electrochemical performance of PtRu@T/GO nanocatalysts

**Figure 4:**
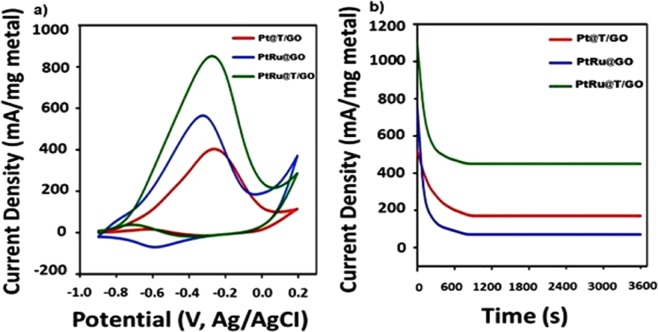
(**a**) Cyclic voltammograms of PtRu@T/GO and Pt@T/GO, PtRu@GO nanocatalysts in nitrogen saturated solution of 0.5 M KOH containing 0.5 M CH_3_OH (Scan rate = 50 mV s^−1^). (**b**) Chronoamperometric curves of PtRu@T/GO and Pt/T/GO, PtRu@GO in 0.5 M KOH nitrogen saturated solution containing 0.5 M CH_3_OH at 0.5 V.

After full characterization of the PtRu@T/GO nanocatalysts, the electrocatalytic activity of these catalysts towards methanol oxidation was studied in Fig. 4a (0.5 M KOH solution saturated with N_2_ gas in 0.5 M CH_3_OH). As can be observed in the forward and backward potential scans, the primary oxidation peak of methanol in PtRu@T/GO was located at nearly −0.28 V, and related peak current density was measured as 876 mA/mg Pt. Also; it is seen that PtRu@T/GO nanocatalysts were 1.82 and 2.32 times more effective compared to the PtRu@GO and Pt/T/GO nanocatalysts, respectively. It can be explained that with the help of T/GO, more active sites can be obtained and give rise to more alcohol oxidation on the surface of the PtRu@T/GO nanocatalysts. This adsorption rate increase can be explained by ascending the active surface area by the aid of T/GO support. Besides, the use of T/GO support in the prepared catalyst prohibits the electrocatalytic reduction in the methanol oxidation reaction and PtRu@T/GO has higher catalytic activity as compared to PtRu@GO, and Pt/T/GO, as shown in Fig. 4a. After obtaining one of the highest currents with the aid of PtRu@T/GO, chronoamperometry (CA) was used for long-term stability tests to compare currents between 1^st^, 50^th^, 100^th^, 200^th^, 500^th^ and 1000^th^ cycles. It was shown that monodisperse PtRu@T/GO nanocatalysts have better catalytic stability and durability compared to the other prepared ones even after 1000 cycles as shown in Fig. S4. As shown in this Fig. S4, the decreasing of the MOR current in PtRu@GO and Pt@T/GO electrodes is much more compared to the one of PtRu@T/GO electrode. The typical CA curves were recorded on PtRu@GO, Pt@T/GO and PtRu@T/GO for MOR are given in Fig. 4b in an electrolyte solution containing methanol (0.5 M), potassium hydroxide (0.5 M) at -0.28 V for 3600 s. The PtRu@T/GO electrode’s current was found to be higher than the other time intervals after 3600 s. The electrodes of PtRu@GO and Pt@T/GO showed a rapid current decay in measurement time compared to the PtRu@T/GO. These findings indicated that the monodisperse PtRu@T/GO electrode shows higher catalytic activity and durability compared to the Pt@T/GO and PtRu@GO electrodes. The electrochemical activities of graphene and graphene oxide supported catalysts used in the literature during methanol oxidation are given in Table 1. In the monometallic case, the oxidation of the platinum decreased because of some poisons like CO, and notably prevented the reaction of methanol oxidation. In PtRu cases, it was thought that ruthenium could react with water, and formed Ru-OH and, strongly bound with CO on Pt, so the PtRu@T/GO and PtRu@GO electrodes had higher catalytic activity and stability for methanol oxidation than Pt@T/GO electrode. Last, but not least, it can also be explained that with the help of T/GO, more active sites were obtained and gave rise to more alcohol oxidation reactions on the surface of the PtRu@T/GO nanomaterials. The electrochemical performance of Pt@T/GO, PtRu@GO, PtRu@T/GO and PtRu and the effects of Pt and Ru contents in the composite on the electrochemical performance in 0.5 M KOH nitrogen saturated solution containing 0.5 M CH_3_OH were examined in detail in Tables S1 and S2. As shown in these tables, PtRu@T/GO is the best catalyst compared to the others and 1:1 ratio of Pt and Ru are the optimum ratio for these prepared nanocatalysts.

**Table 1:** Comparison of electrocatalytic activity of different electrode surfaces in 0.5 M CH_3_OH in 0.5 M KOH at a scan rate of 50 mV·s^−1^.

Electrode	Ipa (mA/mg metal)	Reference
PtRu@T/GO	876.3 ± 5.2	This work
PtRu/TiO_2_-CNF	603	^78^
PtRu/CNF	186	^79^
PtRu/TECNF	516	^80^
TiO_2_-PtRu/C	324	^81^
*PtRu/C_com_	76	^82^

*PtRu/C_com_: The commercial catalyst.

## Conclusions

The current work describes for the controlled synthesis of thiourea functionalized graphene oxide-based PtRu nanocatalysts (PtRu@T/GO) with a series of ultrasonication methods and promises a new catalyst for use in methanol oxidation reactions. Synthesized thiourea (T) based GO (T/GO) was characterized by several morphological techniques and applied as very effective catalysts for the methanol oxidation reactions with the help of the stabilization of T/GO. The method used in this study does not require any expensive systems to prepare natural and environmentally friendly catalysts. PtRu@T/GO indicated an 11-times higher mass activity than PtRu/C_com_, and a 4-times greater than PtRu/CNF. T/GO is the promising support for the PtRu nanocatalysts for the DMFCs and MOR. The long-term stability of the modified electrode with PtRu@T/GO was also performed with the help of CA and it was found that the activity of PtRu@T/GO was higher than the other prepared ones even after 3600 s. The electrodes of modified with PtRu@GO and Pt@T/GO showed a rapid current decay in measurement time compared to the electrode modified with PtRu@T/GO. Besides, it has been observed that even after 1000 cycles, 97.3% of the initial performance was maintained. These findings indicated that the monodisperse modified PtRu@T/GO electrode shows higher catalytic activity and durability compared to the modified Pt@T/GO and PtRu@GO electrodes. PtRu@T/GO nanocatalysts exhibited a highly recyclable, highly efficient and environmentally friendly for methanol oxidation.

## Supplementary information

Supplementary information.
