# Supplementary material for: Monodisperse thiourea functionalized graphene oxide-based PtRu nanocatalysts for alcohol oxidation
Source: Sci Rep. 2020 May 8;10:7811. doi: 10.1038/s41598-020-64885-6 (PMC7210875; doi:10.1038/s41598-020-64885-6)
Supplement: Supplementary file 1 — Supplementary information. [file 41598_2020_64885_MOESM1_ESM.docx]

**SUPPORTING INFORMATION**

**Monodisperse thiourea functionalized graphene oxide-based PtRu nanocatalysts for alcohol oxidation**

**Esra Kuyuldar^a^, Su Selda Polat^a^, Hakan Burhan^a^, Sibel Demiroglu Mustafov^a^, Aysegul Iyidogan^b^, Fatih Sen^a^***

^a^Sen Research Group, Department of Biochemistry, Faculty of Arts and Science, Dumlupınar University, Evliya Çelebi Campus, 43100 Kütahya, Turkey

^b^Department of Chemistry, Faculty of Science and Arts, Gaziantep University, Gaziantep, Turkey

^*^Corresponding author: [fatih.sen@dpu.edu.tr](mailto:fatih.sen@dpu.edu.tr)

Tel:90 274 265 20 31 -37 02 Fax: 90 274 265 20 56

**Materials and Methods**

Platinum (IV) chloride (PtCl_4_), Ruthenium (III) chloride (RuCl_3_) were bought from Merck. Borane dimethylamine, potassium hydroxide (KOH), Nafion solution, tetrahydrofuran, methanol and graphite were bought from Sigma Aldrich. Thiourea functionalized graphene oxide (T@GO) was prepared using by modified Hummer’s method. All the chemicals were used as received. Deionized (DI) water was used for all the experiments. Morphology and structure of the as-synthesized samples were characterized with transmission electron microscopy (JEOL 200 kV TEM). For TEM characterization, Pt/T@GO, PtRu@GO and PtRu/T@GO nanocatalysts were ﬁrst dispersed into ethanol with sonication for several minutes and was then casted on a carbon covered 400-mesh copper grid and dried in ambient condition, and then transferred into the microscope for observation. More than 300 particles were calculated to get the integrated information about the overall distribution of Pt-based catalyst sample. A Panalytical Emperian diffractometer with Ultima+theta–theta high resolution goniometer, having an X-ray generator (Cu Kα radiation, λ = 1.54056 Å) and operating condition of 45 kV and 40 mA, were employed in X-ray diffraction (XRD) analysis. Specs spectrometer was used for X-ray Photoelectron Spectroscopy (XPS) measurements and the X-ray source was Kα lines of Mg (1253.6 eV, 10 mA). Samples were prepared by depositing the catalyst on Cu double-sided tape (3M Inc.). C 1s line at 284.6 eV was chosen as a reference point and all XPS peaks were fitted using a Gaussian function. In O 1s spectrum, binding energies of common chemical states are theoretically ̴ 531 eV, ̴ 533 eV, ̴ 534 eV for C-O, C=O, O-C=O respectively. Also, the binding energy of metal oxide (i.e. platinum oxide) is theoretically ̴ 530 as that is observed in the spectrum PtRu@T/GO nanocatalysts. O 1s spectrum of PtRu@T/GO nanocatalysts (Figure S2) displays that C-O and C=O bonds become mostly prominent while the other oxygen groups have decreased to minimum amounts. The morphological characterization was performed by high-resolution field emission scanning electron microscope (SEM) using a FEI Quanta FEG 250 SEM operating at 10 kV. For FTIR measurements of PtRu@T/GO nanocatalysts, Perkin Elmer Spectrum Two was used. FTIR measurements of PtRu@T/GO nanocatalysts indicated that in the spectrum of thiourea, the absorption bands were exactly match with the literature [1]. In the spectrum of graphene oxide [2], the absorption bands were also very similar with the literature and related peaks. In the spectrum of PtRu@T/GO nanocatalysts, there are some differences in the peaks of epoxy and alkoxy groups compared to GO spectrum. Besides, the stretching vibrations was assigned to the C-S linkage in the region of 800 - 600 cm^-1^. After the addition of thiourea, weak peaks in this region were appeared as a result of nucleophilic addition of thiourea.

**Synthesis of Graphene Oxide (GO)**

The modified Hummer’s method was used in order to prepare graphene oxide from the graphite powder. Briefly; Graphite (1 g) and sodium nitrate (0.5 g) were mixed together and then concentrated sulphuric acid (23 mL) was added under constant stirring. After 1 h, during the gradually addition of KMnO_4_ (3 g) the temperature was kept less than 20 °C to prevent overheating and explosion. This mixture was stirred for 12 h at 35 °C. The resulting solution was diluted by adding of water (500 mL) under vigorous stirring. The suspension was treated with 30% H_2_O_2_ solution (5 mL) to ensure the completion of reaction with KMnO_4_, the resulting mixture was washed sequentially with HCl and H_2_O. Finally, filtration and drying were performed to obtain the graphene oxide sheets.

**Electrochemical measurements**

Electrocatalytic measurements have been carried out using microcomputer-controlled potentiostat/galvanostat (Gamry Interface 1000). A glassy carbon electrode of 3 mm in diameter was carefully polished with alumina powder until a mirror finish was obtained. Then, the electrode was ultrasonically cleaned with methanol and deionized water alternant and dried in the air at room temperature. In order to prepare the catalyst ink, 1 mg of the nanocatalysts was dispersed into 40 µL 0.5 % Nafion solution and 2 mL deionized water and ultrasonically treated for 30 min. 2 µL of the dispersion was coated on the GCE with a micropipette and let it dry in air. A conventional, three-electrode cell consisting of the GC (glassy carbon) working electrode, Pt wire, as counter electrode, and Ag/AgCl reference electrode was used for the cyclic voltammetry (CV) and chronoamperometry (CA) experiment. The CV and CA experiments were performed in 0.5 M KOH solution in the absence and presence of 0.5 M methanol at a scan rate of 50mVs^-1^. The cyclic voltammograms and chronoamperometry of PtRu/T@GO and Pt/T@GO, PtRu@GO nanocatalysts for the electro-oxidation of methanol were recorded at a sweep rate of 50 mVs^-1^in 0.5 M KOH + 0.5 M methanol.


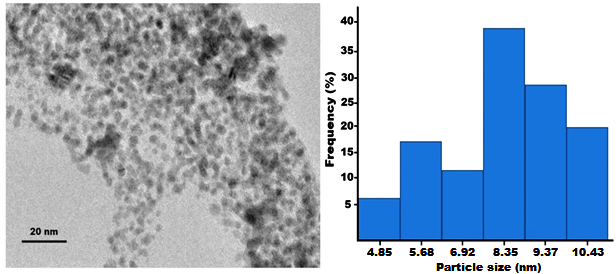


**Figure S1.** TEM image and the diameter size distribution of PtRu/T@GO (after reuse)

**
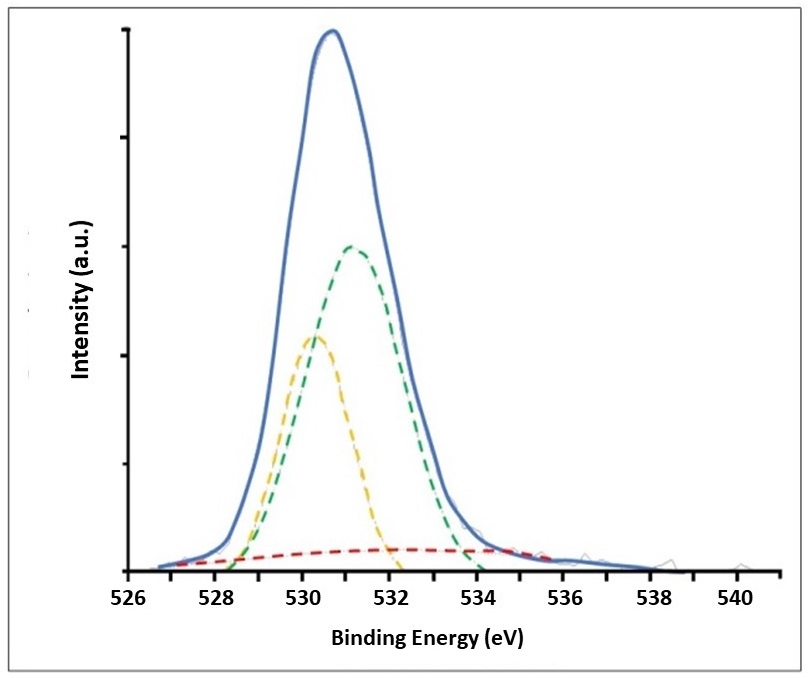
**

**Figure S2.** O1s spectra of PtRu/T@GO

**
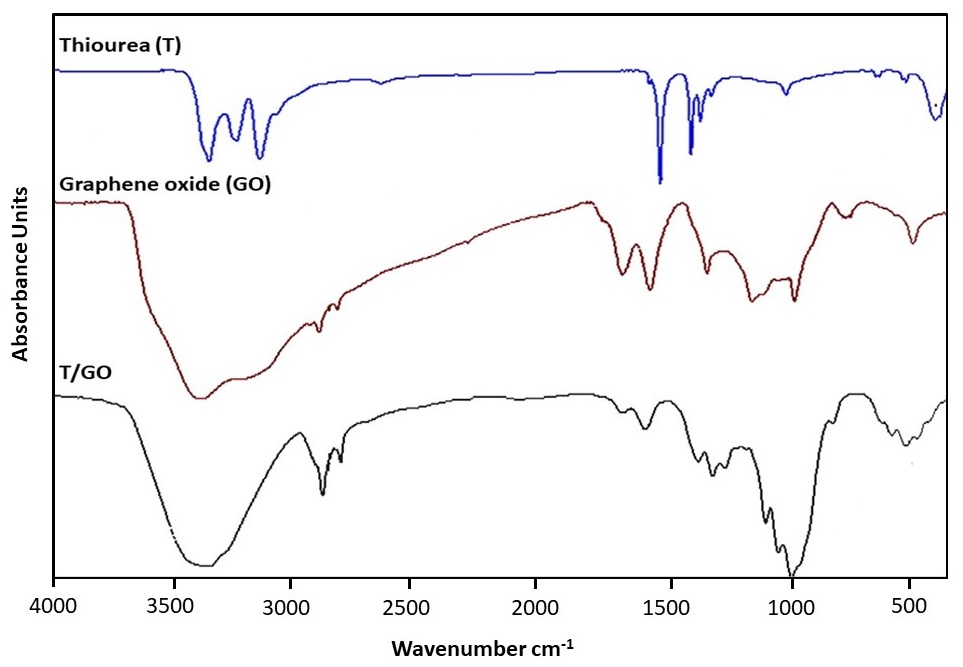
**

**Figure S3.** FTIR of Thiourea (T), graphene oxide (GO), thiourea based graphene oxide (T/GO)

**
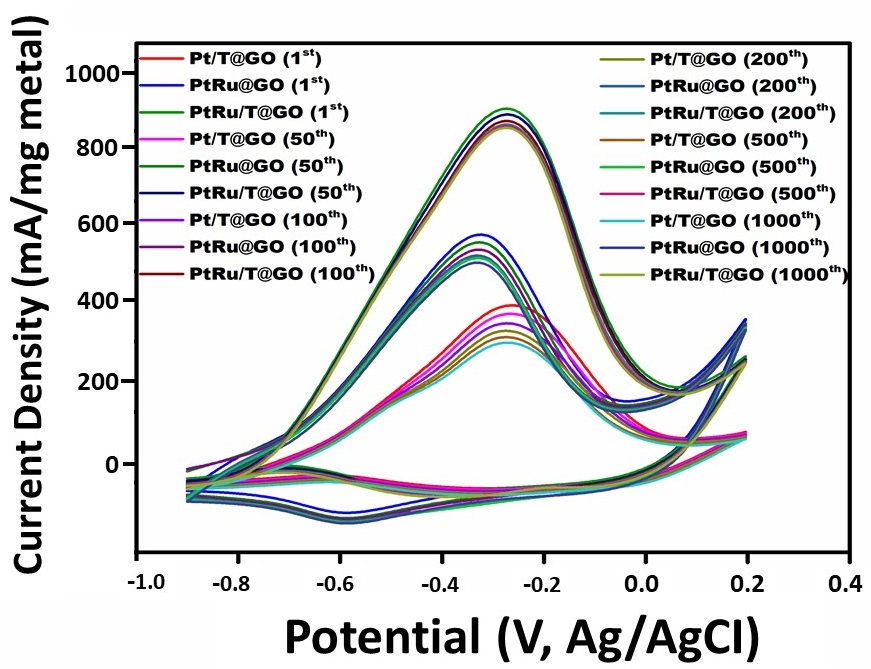
**

**Figure S4.** Catalytic life time measurements of Pt/T@GO, PtRu@GO, PtRu/T@GO in nitrogen saturated solution of 0.5 M KOH containing 0.5 M CH_3_OH at a scan rate of 50 mV s^−1^ at a 1^st^ 50 ^th^, 100 ^th^, 200 ^th^, 500^th^ and 1000^th^ cycle (vs. Ag/AgCl).

**Table S1.** The electrochemical performance of Pt/T@GO, PtRu@GO, PtRu/T@GO and PtRu in nitrogen saturated solution of 0.5 M KOH containing 0.5 M CH_3_OH

| **Material** | **Current Density (mA/mg metal)** | **Anodic Potential (V)** |
| --- | --- | --- |
| **Pt/T@GO** | 318± 3.2 | -0.22 |
| **PtRu@GO** | 513± 2.6 | -0.29 |
| **PtRu/T@GO** | 876.3 ± 5.2 | -0.22 |
| **PtRu** | 597± 4.3 | -0.29 |

**Table S2.** The effects of Pt and Ru contents in the composite on the electrochemical performance in nitrogen saturated solution of 0.5 M KOH containing 0.5 M CH_3_OH

| **Material** | **Current Density (mA/mg metal)** |
| --- | --- |
| **Pt_50_Ru_50_/T@GO** | 876.3 ± 5.2 |
| **Pt_60_Ru_40_/T@GO** | 726 ± 3.3 |
| **Pt_70_Ru_30_/T@GO** | 621 ± 3.1 |
| **Pt_80_Ru_20_/T@GO** | 501 ± 2.4 |
| **Pt_90_Ru_10_/T@GO** | 456 ± 1.6 |

References

**[1]** Stewart, James E. "Infrared absorption spectra of urea, thiourea, and some thiourea‐alkali halide complexes." The Journal of Chemical Physics 26.2 (1957): 248-254.

**[2]** Lu, Jinlin, et al. "Self-assembled platinum nanoparticles on sulfonic acid-grafted graphene as effective electrocatalysts for methanol oxidation in direct methanol fuel cells." Scientific reports 6 (2016): 21530.
